# A random forest based computational model for predicting novel lncRNA-disease associations

**DOI:** 10.1186/s12859-020-3458-1

**Published:** 2020-03-27

**Authors:** Dengju Yao, Xiaojuan Zhan, Xiaorong Zhan, Chee Keong Kwoh, Peng Li, Jinke Wang

**Affiliations:** 1 School of Software and Microelectronics, Harbin University of Science and Technology, Harbin, 150080 China; 2 College of Computer Science and Technology, Heilongjiang Institute of Technology, Harbin, 150050 China; 3 Department of Endocrinology and Metabolism, the First Affiliated Hospital of Harbin Medical University, Harbin, Heilongjiang China; 4 School of Computer Science and Engineering, Nanyang Technological University, Singapore, 639798 Singapore; 5 Department of Software Engineering, Harbin University of Science and Technology, Rongcheng, 264300 China

**Keywords:** Random forest, Variable importance, Feature selection, lncRNA-disease association prediction, Bioinformatics algorithm

## Abstract

**Background:**

Accumulated evidence shows that the abnormal regulation of long non-coding RNA (lncRNA) is associated with various human diseases. Accurately identifying disease-associated lncRNAs is helpful to study the mechanism of lncRNAs in diseases and explore new therapies of diseases. Many lncRNA-disease association (LDA) prediction models have been implemented by integrating multiple kinds of data resources. However, most of the existing models ignore the interference of noisy and redundancy information among these data resources.

**Results:**

To improve the ability of LDA prediction models, we implemented a random forest and feature selection based LDA prediction model (RFLDA in short). First, the RFLDA integrates the experiment-supported miRNA-disease associations (MDAs) and LDAs, the disease semantic similarity (DSS), the lncRNA functional similarity (LFS) and the lncRNA-miRNA interactions (LMI) as input features. Then, the RFLDA chooses the most useful features to train prediction model by feature selection based on the random forest variable importance score that takes into account not only the effect of individual feature on prediction results but also the joint effects of multiple features on prediction results. Finally, a random forest regression model is trained to score potential lncRNA-disease associations. In terms of the area under the receiver operating characteristic curve (AUC) of 0.976 and the area under the precision-recall curve (AUPR) of 0.779 under 5-fold cross-validation, the performance of the RFLDA is better than several state-of-the-art LDA prediction models. Moreover, case studies on three cancers demonstrate that 43 of the 45 lncRNAs predicted by the RFLDA are validated by experimental data, and the other two predicted lncRNAs are supported by other LDA prediction models.

**Conclusions:**

Cross-validation and case studies indicate that the RFLDA has excellent ability to identify potential disease-associated lncRNAs.

## Background

LncRNAs are a category of long non-coding RNAs with transcripts longer than 200 nucleotides [1]. Accumulated evidence demonstrates that lncRNAs are involved in almost all-important biological processes, including gene transcription, cell differentiation, and epigenetic regulation [2–4]. The abnormal regulation of lncRNAs is associated with many complex human diseases, such as various cancers, Alzheimer’s disease, cardiovascular disease and neurodegenerative diseases [5–9]. Therefore, accurately identifying disease-associated lncRNAs is helpful to study the mechanism of lncRNAs in diseases and explore new therapies of diseases.

To reduce the cost of discovering disease-associated lncRNAs by biological experiments, dozens of computational models have been developed to identify disease-associated lncRNAs based on a variety of biological data. At present, LDA prediction models can be classified into three categories. The first type of LDA prediction models is models based on complex network that predict disease-associated lncRNAs by integrating various biological networks [5]. Under the supposition that lncRNAs with analogous function tend to be related to diseases with analogous phenotype, Sun et al. proposed a LDA prediction model named RWRlncD by implementing random walk with restart (RWR) on a LFS network [10]. Under the supposition that the more miRNAs two lncRNAs interacted, the more likely they are related to the analogous diseases, Zhou et al. proposed a LDA prediction model by implementing random walk on a heterogeneous network which integrated the disease similarity network, the miRNA-mediated lncRNA crosstalk network and the experiment-supported LDA network [11]. However, neither of the above methods can be used to new diseases that have not any experiment-supported associated lncRNAs. Chen et al. implemented a LDA prediction model called KATZLDA by integrating the Gaussian interaction profile kernel similarity (GIPKS) and semantic similarity of diseases, the expression profiles and functional similarity of lncRNAs, and the experiment-supported LDAs [12]. In addition, Chen et al. developed an improved RWR based LDA prediction model (IRWRLDA), which set the initial probability vector of RWR by combining the lncRNA expression similarity with the DSS [13]. Both of the above methods can be used to new diseases that have not any experiment-supported associated lncRNAs. Moreover, Yu et al. implemented a bi-random walks based LDA prediction model (BRWLDA) [14]. Gu et al. developed a random walk based LDA prediction model on global network (GrwLDA) [15]. Zhang et al. constructed a flow propagation algorithm based LDA prediction model (LncRDNetFlow) [16]. Xiao et al. proposed a paths of fixed lengths based LDA prediction model (BPLLDA) [17]. Ping et al. inferred potential LDAs by an experiment-supported LDA network [18]. Fan et al. implemented a RWR based LDA prediction model (IDHI-MIRW) by combining the positive pointwise mutual information with multiple heterogeneous information [19]. Liu et al. constructed LDA prediction model based on label propagation algorithm on weighted network (NBLDA) [20]. Li et al. developed a local random walk based LDA prediction model (LRWHLDA) [21]. Sumathipala et al. developed a network diffusion based LDA prediction model by integrating the protein-disease, protein-lncRNA and protein-protein associations [22]. Zhang et al. developed a DeepWalk based LDA prediction model by integrating the miRNA-disease, lncRNA-disease, and miRNA-lncRNA associations [23]. Xie et al. implemented a similarity kernel fusion based LDA prediction model (SFK-LDA) by fusing the DSS and cosine similarity, and the lncRNA expression similarity and cosine similarity [24].

The second type of LDA prediction models predict disease-associated lncRNAs based on the expression levels and regulation relationships between disease-associated genes/miRNAs and lncRNAs [5]. Liu et al. implemented the first LDA prediction model not depending on the known LDAs by combining experiment-validated disease-associated genes with the gene/lncRNA expression profiles [25]. However, this model cannot be used for diseases that have not any experiment-validated associated genes. Li et al. proposed a genome location based model for screening human vascular disease-associated lncRNAs [26]. However, this model is invalid for lncRNAs that have no neighbour genes. Chen developed a hypergeometric distribution based LDA prediction model (HGLDA) by combining the LMI with MDAs [27]. HGLDA has a reliable performance for LDA prediction, but it cannot be used for lncRNAs that have not any experiment-supported interacted miRNAs [5]. Wang et al. developed a sequence based LDA prediction model (LncDisease) using the known lncRNA-miRNA crosstalk [28]. However, because the predicted miRNA-lncRNA interactions have high false negative and false positive, the performance of LncDisease is limited. Moreover, Cheng et al. developed information flow modelling based LDA prediction model (IntNetLncSim) by combining lncRNA-associated transcriptional information with post-transcriptional information [29]. Wang et al. developed a competing endogenous RNAs (ceRNAs) based LDA prediction model (DisLncPri) by mapping lncRNAs to their functional genomics context [30]. Fu et al. proposed a matrix factorization based LDA prediction model (MFLDA) by decomposing multiple data matrices into low-rank matrices to identify their interior structure [31]. Ding et al. developed an lncRNA-disease-gene tripartite graph based LDA prediction model (TPGLDA) by combining the gene-disease associations with the LDAs [32]. Lu et al. developed an inductive matrix completion based LDA prediction model (SIMCLDA) by integrating the gene-disease, lncRNA-disease and gene-gene associations [33]. Wang et al. implemented a weighted matrix factorization based LDA prediction model (WMFLDA) by pre-setting weights to various association matrices among genes, lncRNAs and diseases and decomposing these matrices into low-rank matrices [34].

The third type of LDA prediction models predict disease-associated lncRNAs based on various machine learning algorithms [5]. Under the supposition that analogous diseases tend to be related to analogous lncRNAs, Chen et al. proposed a Laplacian regularized least squares based LDA prediction model (LRLSLDA) by combining the experiment-supported LDAs with the lncRNA expression profiles [35], which is the first computational model in this field. LRLSLDA is a semi-supervised machine learning model not needing negative samples, but how to optimize model parameters remains a problem. Later, Chen et al. implemented a new LDA prediction model named LRLSLDA-LNCSIM by combining the functional, expression and GIPKS of lncRNAs with the semantic and GIPKS of diseases [36]. LRLSLDA-LNCSIM improves the performance of LRLSLDA. Furthermore, Huang et al. proposed an improved LFS model (ILNCSIM) by using the topological characteristics of disease DAGs (directed acyclic graphs) [37]. In addition, Zhao et al. implemented a naïve Bayesian classifier based lncRNA-cancer association prediction model by integrating the genome, transcriptome and regulome data [38]. Lan et al. implemented a bagging SVM classifier based LDA prediction model (LDAP) by combining the disease similarity with the lncRNA similarity [39]. Yu et al. proposed a naïve Bayesian classifier based collaborative filtering LDA prediction model (CFNBC) by integrating the lncRNA-disease, miRNA-disease and lncRNA-miRNA associations [40]. Guo et al. developed a rotating forest and neural network based LDA prediction model (LDASR) by combining the lncRNA GIPKS with the DSS and GIPKS [41]. Chen et al. implemented a support vector machine based LDA prediction model (ILDMSF) by integrating the lncRNA-gene interactions, the lncRNA-disease associations and the DSS [42]. Guo et al. implemented a random forest classifier based model for inferring novel associations among various bimolecular by constructing a molecular association network based on the known associations among diseases, proteins, miRNAs, lncRNAs and drugs [43]. Latterly, Xuan et al. proposed a series of convolutional neural network based LDA prediction models, including CNNLDA [44], GCNLDA [45], CNNDLP [46] and LDAPred [47]. CNNLDA learned the global and attention characteristics of lncRNA-disease pairs using convolutional neural networks by integrating the DSS, the LFS, and the lncRNA-disease, miRNA-disease and lncRNA-miRNA associations [44]. GCNLDA learned the local and network characteristics of lncRNA-disease pairs using convolutional neural network, graph convolutional network and convolutional auto-encoder by combining multiple associations among diseases, miRNAs and lncRNAs [45]. CNNDLP learned the network and attention characteristics of lncRNA-disease pairs using convolutional neural network and convolutional auto-encoder by integrating various associations, interactions and similarities among miRNAs, lncRNAs and diseases [46]. LDAPred implemented LDA prediction using convolutional neural network and information flow propagation by integrating various associations, interactions, similarities and topology among miRNAs, lncRNAs and disease [47]. These four methods have better performance for LDA prediction, but they all need to adjust many model parameters.

Inspired by previous works [44, 48], we implemented a random forest and feature selection based LDA prediction model (RFLDA). First, the RFLDA represented lncRNA-disease pairs by a high-dimensional feature vector that integrated the DSS, the LFS, the experiment-supported LDAs and MDAs, and the miRNA-lncRNA interactions. Then, the RFLDA chose more useful features based on the variable importance score of random forest to represent lncRNA-disease samples. Finally, the RFLDA employed a random forest regression model trained on low-dimensional feature space to score potential LDAs. The AUC and AUPR under 5-fold cross-validation demonstrate that the RFLDA has better performance than several outstanding LDA prediction models. Moreover, case studies on three cancers indicate that the RFLDA has excellent ability to identify disease-associated lncRNAs.

## Results

### Feature selection

**Fig. 1:**
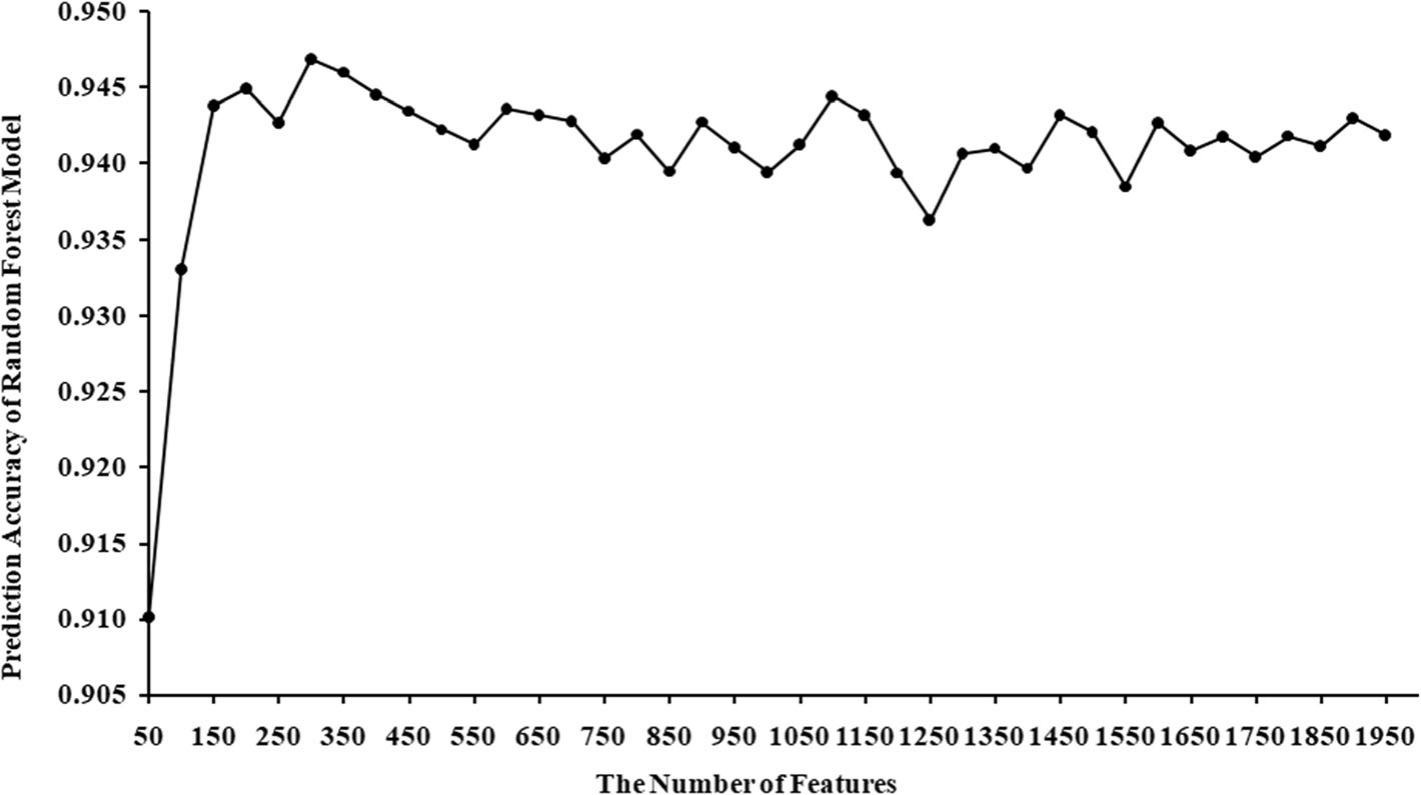
Prediction accuracy of random forest models on different training sample set consisting of different number of features

To determine how many features should be used to train random forest regression model, we studied the prediction accuracy of models on different training sample sets by 10-fold cross-validation. Specially, we chose the top 50, 100 … 1900 and 1950 most important features (with the largest variable importance scores) to train random forest models in turn and calculated their prediction accuracy. The prediction accuracy under 10-fold cross-validation of random forest models trained using different number of features is shown in Fig. 1. As one can see from Fig. 1, the prediction accuracy of random forest models gradually increases with more features being added into training sample set, and achieves the largest value, 0.947, on training sample set consisting of the top 300 most important features. Therefore, in this work, we utilized the top 300 most important features to train the RFMDA model and evaluate its performance. The variable importance scores of all 1952 features are listed in Table S1, and the prediction accuracy of all random forest models on different training samples is listed in Table S2.

### Performance measures

The AUC and AUPR under 5-fold cross-validation are calculated to evaluate the ability of different LDA prediction models. The 2697 experiment-supported LDAs are considered as positive samples. The 2697 randomly selected lncRNA-disease pairs not validated by experiments are considered as negative samples. All lncRNA-disease pairs not validated by experiments are taken as unlabelled samples. For 5-fold cross-validation, all positive and negative samples are evenly divided into 5 parts. In each cross-validation, four parts of positive samples and negative samples are used for training random forest model in turn, and the leftover positive samples and all unlabelled samples are used as testing samples. Then, a random forest regression model is trained to score testing samples. As a result, each test sample (lncRNA-disease pair) is given a score that represented the likelihood that the lncRNA and disease of this sample are associated. Next, all test samples are sorted in descending by their prediction scores. On this basis, we calculated the false positive rate (*FPR*) and the true positive rate (*TPR*) with different thresholds. The *FPR* represents the proportion of the real negative samples in predicted positive samples (test samples that are ranked before the given threshold) to all negative samples. The *TPR* represents the proportion of the real positive samples in predicted positive samples (test samples that are ranked before the given threshold) to all positive samples. The *TPR* and the *FPR* can be calculated by eq. 1 and eq. 2, respectively.

1 $$ TPR=\frac{TP}{TP+ FN} $$

2 $$ FPR=\frac{FP}{FP+ TN} $$

where, *TP* (true positive) means that a positive sample is correctly predicted as positive sample; *FN* (false negative) means that a positive sample is incorrectly predicted as negative sample; *FP* (false positive) means that a negative sample incorrectly predicted as positive sample; *TN* (true negative) means that a negative sample is correctly predicted as negative sample. Using *TPR* as vertical axis and *FPR* as horizontal axis, the receiver operating characteristic (ROC) curve is drawn, and the AUC is calculated to evaluate the prediction ability of different LDA prediction models [49]. The larger the AUC is, the better the model is.

Because the number of negative samples (unconfirmed LDAs) is much larger than the number of positive samples (experiment-supported LDAs), it is seriously imbalanced between them. Therefore, we also draw the precision-recall (PR) curve and calculate the AUPR to evaluate the prediction ability of different LDA prediction model [50]. The *Precision* means the percentage of the accurately predicted positive samples in all predicted positive samples, and the *Recall* means the percentage of the accurately predicted positive samples in all real positive samples. The *Precision* and the *Recall* can be calculated by eq. 3 and eq. 4, respectively.

3 $$ Precision=\frac{TP}{TP+ FP} $$

4 $$ Recall=\frac{TP}{TP+ FN} $$

Giving that 5-fold cross-validation, we adopt the average values of AUCs/AUPRs in five folds to evaluate the performance of different LDA prediction models. Moreover, to get reliable results, we repeated each experiment 10 times and computed the average value of 10 times experiments to be the final evaluation results.

### Performance comparison with other prediction models

**Fig. 2:**
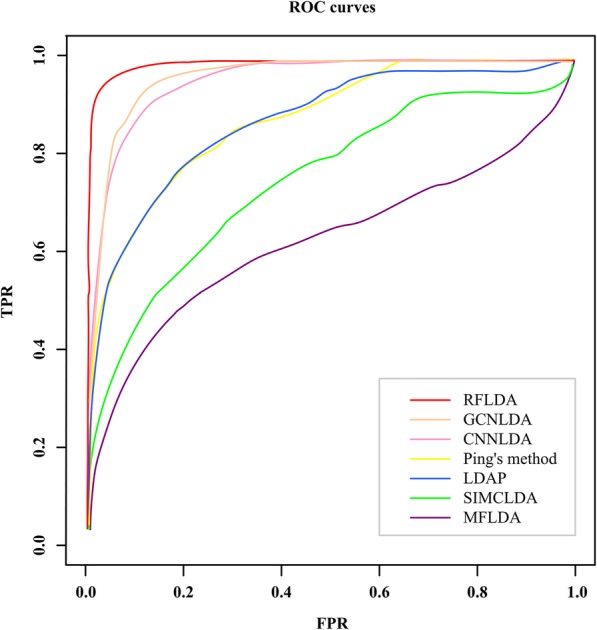
The ROC curves of different LDA prediction models

To show the prediction ability of the RFLDA, we compare it with several excellent LDA prediction models, such as SIMCLDA [33], Ping’s method [18], MFLDA [31], LDAP [39], CNNLDA [44], and GCNLDA [45]. The AUCs and AUPRs of all LDA prediction models are shown in Table 1. The ROC curves of different LDA prediction models are shown in Fig. 2. The AUCs and AUPRs of the RFLDA in each cross-validation are listed in Table S3.

**Table 1:** The AUCs and AUPRs of different LDA prediction models

Algorithm	AUC	AUPR
SIMCLDA	0.746	0.095
Ping’s Method	0.871	0.219
MFLDA	0.626	0.066
LDAP	0.863	0.166
CNNLDA	0.952	0.251
GCNLDA	0.959	0.223
RFLDA	**0.976**	**0.779**

As one can see, the RFLDA achieves AUC of 0.976 (±0.0002) on all tested 412 diseases, which is higher than all other methods involved in the comparison. It outperforms SIMCLDA by 31%, Ping’s method by 12%, MFLDA by 56%, LDAP by 13%, CNNLDA by 3% and GCNLDA by 2%. Moreover, RFLDA achieves AUPR of 0.779 (±0.0297) on all tested 412 diseases, which is also higher than all other methods involved in the comparison. Specifically, it outperforms SIMCLDA by 720%, Ping’s method by 256%, MFLDA by 1080%, LDAP by 369%, CNNLDA by 210% and GCNLDA by 249%. The comparison results indicate that the RFLDA has excellent ability of LDA prediction. It should be noted that the AUCs and AUPRs of other six models except RFLDA in Table 1 are derived from Xuan et al.’s work [44, 45].

### Case studies

To further show the ability of the RFLDA to identify new disease-associated lncRNAs, case studies on stomach cancer, lung cancer and colon cancer are constructed. First, we trained the RFLDA on a sample set that did not contain any validated associations between lncRNAs and the investigated diseases. Here, all known lncRNA-disease associations from Fu et al.’s previous work [31], except for the investigated diseases, were taken as positive samples to training random forest prediction model, and all unconfirmed lncRNA-disease pairs were used as test samples. Then, we scored and sorted all unconfirmed lncRNA-stomach/lung/colon cancer samples. Finally, we validated the predicted lncRNAs associated with stomach/lung/colon cancer by the records in the Lnc2Cancer (v2.0) [51], LncRNADisease (v2.0) [52], and published literature [53–56]. The Lnc2Cancer is a manually managed lncRNA-cancer association database, which stores the 4986 experiment-validated associations between 165 cancers and 1614 lncRNAs. The LncRNADisease is a manually managed lncRNA-disease association database, which stores the 10,564 experiment-validated LDAs and the 195,395 predicted LDAs by excellent LDA prediction methods. As a result, the top 15 predicted lncRNAs associated with the three cancers by the RFLDA are shown in Table 2, respectively.

**Table 2:** The candidate lncRNAs associated with stomach cancer, lung cancer and colon cancer

Diseases	Rank	LncRNA	Evidence
Stomach Cancer	1	HULC	C & D
	2	MIR17HG	L& D*
	3	TUG1	C & D
	4	HOTTIP	C & D
	5	NEAT1	C & D
	6	WT1-AS	C & D
	7	MALAT1	C & D
	8	TP53COR1	C
	9	HNF1A-AS1	C & D
	10	SPRY4-IT1	C & D
	11	MIR155HG	D*
	12	KCNQ1OT1	D
	13	NPTN-IT1	C & D
	14	BCYRN1	C & D*
	15	CDKN2B-AS1	C & D
Lung Cancer	1	HOTTIP	C & D
	2	HULC	L& D*
	3	SPRY4-IT1	C & D
	4	PCAT1	C & D
	5	TP53COR1	C & D
	6	SOX2-OT	C & D
	7	IGF2-AS	C & D*
	8	SPRY4-IT1	C & D
	9	KCNQ1OT1	C & D
	10	PRNCR1	L& D*
	11	MIR100HG	D*
	12	TUSC7	C & D*
	13	PVT1	C & D
	14	GHET1	C & D*
	15	BCYRN1	C & D
Colon Cancer	1	HOTTIP	C & D
	2	HULC	C & D
	3	GAS5	C & D
	4	MIR17HG	L& D*
	5	CDKN2B-AS1	C & D
	6	TUG1	C & D
	7	PVT1	C & D
	8	BANCR	C & D
	9	AFAP1-AS1	C & D
	10	XIST	C & D
	11	HNF1A-AS1	C & D
	12	UCA1	C & D
	13	NEAT1	C & D
	14	SPRY4-IT1	C & D
	15	BCYRN1	C & D

“C” means that the candidate lncRNA is supported by the Lnc2Cancer database. “D” means that the candidate lncRNA is supported by the experiment-validated data in the LncRNADisease database. “D*” means that the candidate lncRNA is supported by the predicted data in the LncRNADisease database. “L” means that the candidate lncRNA is supported by the published literature.

As one can see from Tables 2, 14 of the top 15 predicted stomach cancer-associated lncRNAs by the RFLDA are supported by the experimental data or the published literature, and the remaining one (MIR155HG) is supported by other LDA prediction models. Specially, MIR17HG has been shown to be abnormaly regulated in stomach cancer in published literature [53]. In addition, 14 of the top 15 predicted lung caner-associated lncRNAs by the RFLDA are supported the experimental data or the publised literature, and the remaining one (MIR100HG) is supported by other LDA prediction models. Specially, HULC has been discovered to be dysregulated in lung cancer in published literature [54]; Cheng et al. discovered that PRNCR1 could upregulate HEY2 by competitively bind miR-448 to promote tumor progression in non-small cell lung cancer [55]. Moreover, all top 15 predicted colon cancer-associated lncRNAs by the RFLDA are supported by the experimental data or the published literature. Specially, Xu et al. discovered that MIR17HG was upregulated in colorectal cancer tissue and could promote metastasis and tumorigenesis of colorectal cancer cells [56]. In summary, 43 of the top 45 predicted lncRNAs associated with the three cancers by the RFLDA are supported by the experimental data in the Lnc2Cancer database, the LncRNADisease database or the published literatures, and the remaining 2 lncRNAs are supported by other LDA prediction models. Therefore, case studies show that the RFLDA has excellent ability for LDA prediction.

Beside the three diseases analyzed in case studies, the RFLDA is also used to predict the potential associated lncRNAs for other 409 diseases in this research. The predicted top 50 lncRNAs associated with all 412 diseases by the RFLDA are listed in Table S4, which contains three columns: name of disease, name of lncRNA, and the association score predicted by the RFLDA.

## Discussion

Increased evidence suggests that dysregulation of some lncRNAs are involved in many complex human diseases. Accurately discovering lncRNAs associated with diseases is helpful to explore the pathogenesis and appropriate treatment options of diseases. Due to the high cost of experimental method for identifying disease-associated lncRNAs, researchers have proposed a series of computational model for LDA prediction. However, most of the existing models ignore the interference of noisy and redundancy information among multiple data resources. To improve the performance of LDA prediction models, we developed a random forest and feature selection based LDA prediction model (RFLDA). The AUC and AUPR under 5-fold cross-validation show that the RFLDA are better than several excellent LDA prediction models including SIMCLDA, Ping’s method, MFLDA, LDAP, CNNLDA and GCNLDA. Moreover, case studies on three cancers show that the RFLDA has excellent ability to identify potential disease-associated lncRNAs.

We identify the following reasons why the RFLDA can achieve better performance. First, the RFLDA integrates multiple types of biological data including the experiment-supported LDAs, the functional similarity of lncRNAs, the semantic similarity of diseases, the experiment-supported MDAs, and the interactions between lncRNAs and miRNAs. Second, as an excellent machine learning algorithm, random forest has high accuracy and robustness. By combining random re-sampling and weak classifier assembling, random forest can implement the unbiased estimator for generalization error and good generalization performance. Third, the variable importance score of random forest takes into account not only the effect of an individual feature on the sample prediction but also the joint effect of multiple features on sample prediction. Therefore, the feature selection method based on random forest variable importance score can effectively identifying the most important features for sample prediction.

There are some limitations in RFLDA model. First, RFLDA predicts LDA using the supervised random forest algorithm, which requires both positive and negative samples. However, it is almost unrealistic to obtain the reliable negative samples for LDA prediction. The method of randomly selecting negative samples may influence the prediction performance of RFLDA. Besides, limitation of knowledge about diseases, lncRNA, and miRNAs constrain the prediction performance of RFLDA. Finally, there are many excellent association prediction computational models in various fields of computational biology, such as miRNA/lncRNA-disease association prediction [57–62], drug-target interaction prediction [63], and synergistic drug combination prediction [64]. These association prediction models would provide valuable insights into the development of new lncRNA-disease association prediction. Therefore, we will further improve the performance of LDA prediction model in the future by integrating more biological data and the most advanced algorithm idea of different association prediction.

## Conclusion

Accurately identifying disease-associated lncRNAs is helpful to explore the functionary mechanism of lncRNAs in diseases. Predicting disease-associated lncRNAs by computational methods is an efficient mean. In this work, we developed a random forest and feature selection based LDA prediction model by integrating the LFS, the DSS, the experiment-supported LDAs, the experiment-supported MDAs, and the miRNA-lncRNA interactions. The feature selection based on the variable importance score of random forest was implemented to choose more useful features to train LDA prediction model. The random forest regression model was trained to predict potential LDAs. Cross-validation and case study show that the RFLDA outperforms several excellent LDA prediction models. Therefore, we anticipate that the RFLDA can provide help for the mechanism studies of lncRNAs in diseases in the future.

## Methods

### Datasets for LDA prediction

The datasets used for constructing the RFLDA model include the experiment-supported LDAs and MDAs, and the LMI. All these kinds of datasets come from Fu et al.’s previous study on LDA prediction [31]. Specifically, the 2697 experiment-supported LDAs are originally collected from the Lnc2Cancer [51], LncRNADisease [52] and GeneRIF [65] database. In addition, the 13,562 experiment-supported MDAs originally come from the HMDD (v2.0) [66] database. Moreover, the 1002 LMI originally come from starBase [67] database. In summary, all these datasets cover 240 lncRNAs, 495 miRNAs and 412 diseases.

### Representation of LDA and MDA

The LDAs are represented by an 240 × 412 adjacency matrix *LD* (Fig. 1a). According to the 2697 experiment-supported LDAs, the value of the element of the *LD*, *LD*(*l*(*i*), *d*(*j*)), is set as 1 if lncRNA *l*(*i*) has been confirmed to be related to disease *d*(*j*), otherwise 0. Similarly, the MDAs are represented by an 495 × 412 adjacency matrix *MD* (Fig. 1c). According to the 13,562 experiment-supported MDAs, the value of the element of the *MD*, *MD*(*m*(*i*), *d*(*j*)), is set as 1 if miRNA *m*(*i*) has been validated to be related to disease *d*(*j*), otherwise 0.

### Representation of DSS

Under the supposition that two analogous diseases tend to be related to analogous lncRNAs, disease similarities are integrated into the RFLDA for LDA prediction. Disease Ontology (DO) [68] adopted a type of semantic associations (‘IS_A’ relationship) to represent the association between disease terms. According to ‘IS_A’ relationship between disease terms, we can use a DAG to represent a disease *D*. In the DAG(*D*), the vertexes represent disease *D* and all of its ancestral disease terms, and each of the directed edges represents an ‘IS_A’ relationship linking two diseases. Under the supposition that the more common disease terms two diseases share, the more similar they are, the DSS can be calculated according to their DAGs. Here, we calculate disease semantic similarities by Wang et al.’s method [69]. Specifically, the semantic value of a disease *D*, *DV*(*D*), is calculated by eq. 5.

5 $$ DV(D)={\sum}_{d\in S(D)}{DC}_D(d) $$

where *S*(*D*) represents the node set of *DAG(D)*, *DC*_*D*_(*d*) represents the contribution degree of a disease *d* in *DAG(D)* to disease *D*’s semantic value and is calculated by eq. 6.

6 $$ \left\{\begin{array}{c}{DC}_D(d)=1\kern19.5em if\ d=D\\ {}{DC}_D(d)=\mathit{\max}\left\{\Delta  \ast {DC}_D\left({d}^{\prime}\right)|{d}^{\prime}\in children\ of\ d\right\}\kern1.75em if\ d\ne D\end{array}\right. $$

Where, *∆* is the attenuation coefficient of semantic contribution and is equal to 0.5 by default. As can be seen from eq. 6, the contribution degree of disease *D* to itself is equal to 1, while the contribution degree of other diseases to disease *D* is reduced as the length between them increases. Then, the DSS between *d*(*i*) and *d*(*j*), *DS*(*d*(*i*), *d*(*j*)), is calculated by eq. 7.

7 $$ DS\left(d(i),d(j)\right)=\frac{\sum_{d\in S\left(\mathrm{d}(i)\right)\cap S\left(\mathrm{d}(j)\right)}\left({DC}_{\mathrm{d}(i)}(d)+{DC}_{\mathrm{d}(j)}(d)\right)}{DV\left(d(i)\right)+ DV\left(d(j)\right)} $$

In this work, we calculate disease semantic similarities between 412 diseases using DincRNA online toolkit [70], and represent them by a 412 × 412 similarity matrix *DD* (Fig. 1b), where the value of the element of the *DD*, *DD*(*d*(*i*), *d*(*j*)), represents the DSS of *d*(*i*) and *d*(*j*).

### Representation of LFS

Based on the supposition that two lncRNAs associated with analogous diseases may have analogous functions, the LFS can be computed according to diseases associated with them. Here, we calculate lncRNA functional similarities by Chen et al.’s method [36]. Here, we assume that lncRNA *l*(*a*) is related to a group of diseases *DG*(*a*) = {*d*(*a*1), *d*(*a*2), …, *d*(*am*)}, and lncRNA *l*(*b*) is related to a group of diseases *DG*(*b*) = {*d*(*b*1), *d*(*b*2), …, *d*(*bn*)}, then the LFS between *l*(*a*) and *l*(*b*), denoted as *LS*(*l*(*a*), *l*(*b*)), can be obtained by calculating the similarity between *DG*(*a*) and *DG*(*b*) by eq. 8.

8 $$ LS\left(l(a),l(b)\right)=\frac{\sum_{i=1}^m\begin{array}{c}\max \\ {}1\le j\le n\end{array}\left( DS\Big(d(ai),d(bj)\Big)\right)+{\sum}_{j=1}^n\begin{array}{c}\max \\ {}1\le i\le m\end{array}\left( DS\Big(d(bj),d(ai)\Big)\right)}{m+n} $$

Where *DS*(*d*(*ai*), *d*(*bj*)) is the semantic similarity between the disease *d*(*ai*) in *DG*(*a*) and the disease *d*(*bj*) in *DG*(*b*); *m* and *n* represent disease numbers of the *DG*(*a*) and the *DG*(*b*), respectively. In this work, the LFS is represented by a 240 × 240 similarity matrix *LL* (Fig. 1a), where the value of the element of the *LL*, *LL*(*l*(*i*), *l*(*j*)), represents the LFS of *l*(*i*) and *l*(*j*).

### Representation of LMI

Cumulative evidence indicates that the lncRNAs can interact with the corresponding miRNAs and perform biological functions together with these miRNAs [71]. Therefore, the LMI are integrated into the RFLDA model for lncRNA-disease association prediction, which is represented by an 240 × 795 adjacency matrix *LM* (Fig. 1c). According to 1002 LMI extracted from starBase database, the value of the element of the *LM*, *LM*(*l*(*i*), *m*(*j*)), is set as 1 if there is an interactions between miRNA *m*(*j*) and lncRNA *l*(*i*), otherwise *0*.

### Construction of the RFLDA model

**Fig. 3:**
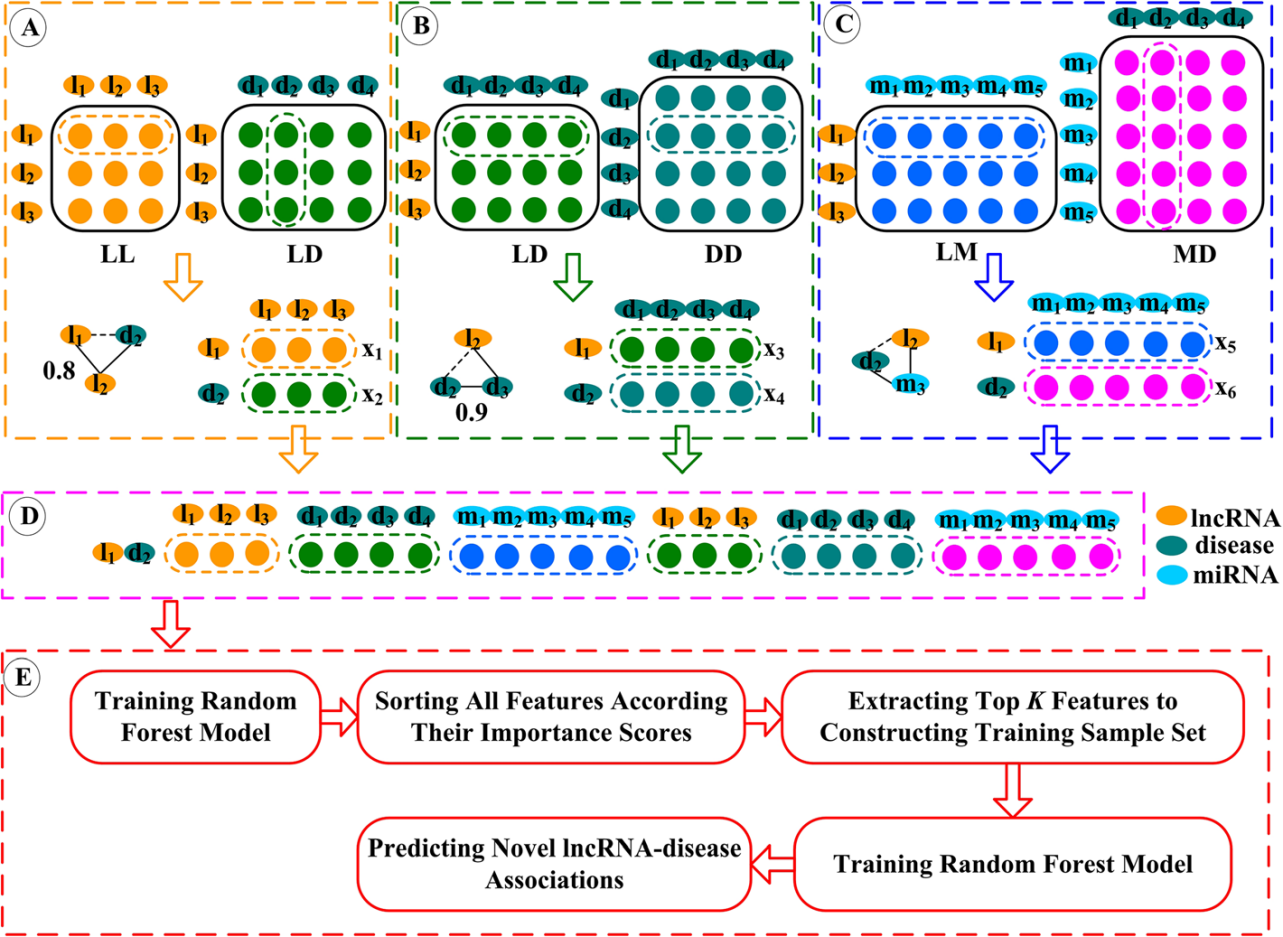
Flowchart of constructing the RFLDA model

The RFLDA model is constructed by four steps (see Fig. 3): (1) sample representation; (2) training sample set construction; (3) feature selection; (4) random forest construction and LDA prediction. Next, we introduce the process of constructing RFLDA in detail.

### Sample representation

In our RFLDA model, we take an lncRNA-disease pair as a sample. By integrating the functional similarity of lncRNAs (Fig. 2a), the experiment-supported associations between lncRNAs and diseases (Fig. 2a), the semantic similarity of diseases (Fig. 2b), the interactions between lncRNAs and miRNAs (Fig. 2c), and the experiment-supported associations between miRNAs and diseases (Fig. 2c), we use an 1147-dimensional feature vector to represent an lncRNA and a disease respectively. Therefore, a sample can be represented by a 2294-dimensional feature vector (Fig. 2d), denoted as *F*, which can be represented by eq. 9 in detail.

9 $$ F=\left({f}_1,{f}_2,\cdots, {f}_{240},{f}_{241},\cdots, {f}_{652},{f}_{653},\cdots, {f}_{1147},{f}_{1148},\cdots, {f}_{1387},{f}_{1388},\cdots, {f}_{1799},{f}_{1800},\cdots, {f}_{2294}\right) $$

Where (*f*_1_, *f*_2_, ⋯, *f*_240_) represents the 240 lncRNA-lncRNA similarities, (*f*_241_, ⋯, *f*_652_) represents the 412 lncRNA-disease associations, (*f*_653_, ⋯, *f*_1147_) represents the 495 lncRNA-miRNA interactions, (*f*_1148_, ⋯, *f*_1387_) represents the 240 disease-lncRNA associations, (*f*_1388_, ⋯, *f*_1799_) represents the 412 disease-disease similarities, and (*f*_1800_, ⋯, *f*_2294_) represents the 495 disease-miRNA associations. Finally, we normalized *f*_*i*_ to *f*_*i*_^′^ by eq. 10.

10 $$ {f_i}^{\prime }=\frac{f_i-{f}_{m\mathrm{i}n}}{f_{max}-{f}_{min}} $$

Where *f*_*max*_ and *f*_*min*_ were the maximum and the minimum of *f*_*i*_ (*i* = 1, 2… 2294) in all samples.

### Training sample set construction

First, the 2697 experiment-supported LDAs were used as positive samples, and all lncRNA-disease pairs not validated by experiments were taken as unlabelled samples. In addition, the 2697 randomly selected unlabelled samples were taken as negative samples. Finally, all negative samples and positive samples were combined as training samples.

### Feature selection based on variable importance score of random forest

Random forest (RF) [72] is an integrated machine learning algorithm proposed by Breiman in 2001, which combines Bagging technology and random subspace method to realize randomness and diversity between base classifiers. First, RF randomly selects multiple samples from the original sample set with replacement using the Bootstrap technology. Then, it constructs a decision tree on each Bootstrap sample set. In the process of training the decision tree, it randomly selects a feature from a feature set for node splitting at each node by random subspace method. Finally, it combines multiple decision trees and determines the classification or prediction results by majority vote. Compared with other machine learning algorithms, RF has many advantages: (1) it can process a variety of data types, including qualitative data or quantitative data; (2) it provides a measure of the variable importance, which provides an easy way to understand the relative importance of features for classification or prediction model; (3) it has high classification accuracy; (4) it has good robustness for noise data and data with missing values; (5) it has ability to analyse complex interactions between features; (6) it has a fast learning speed with the increase of the number of input variables [73]. In recent years, RF has been widely used in a variety of classification and prediction problems, such as DNA binding protein recognition [74], genetic polymorphism recognition [75], prediction of medium and long-term chaotic regions of protein sequences [76], differential expression analysis of microarray data [77], miRNA-disease association prediction [78], etc.

In this work, because each sample has 2294 features, it contains a lot of noisy and redundant information. To improve the prediction performance while reduce the computational cost, we performed feature selection before training LDA prediction model. First, we removed 312 features whose values were 0 in all samples. As a result, 1952 features were preserved. Then, we implemented feature selection according to the variable importance score of random forest which is calculated by the average decrement of the classification accuracy of random forest model before and after small perturbation of the variable in OOB (outside of bag) [77]. Because the variable importance score of random forest takes into account not only the impact of each individual variable on the response variable but also the interaction of multiple variables on the response variable, it is often used to rank features to select more important features [78]. In the RFLDA, we firstly trained a random forest model on the original training sample set consisting of 1952 features and computed variable importance scores of all features; then, we ranked 1952 features in descending order according to their scores; finally, we selected the top 300 features with the highest variable importance scores to represent the training samples. To get reliable results, we calculated the variable importance scores for all features 10 times, and selected important features according to the average variable importance score of each feature.

### Random forest construction and LDA prediction

In the last step of the RFLDA, we firstly constructed a random forest regression model using the training sample set consisting of the top 300 most important features by running randomForest package on *R* platform. In the training sample set, each positive sample was labelled as 1 while each negative sample was labelled as 0. Then, we used the random forest prediction model to score unconfirmed lncRNA-disease pairs. The larger the score of an lncRNA-disease pair, the more likely the lncRNA and the disease are associated. It should be noted that two main parameters in random forest algorithm, the *mtry* and the *ntree*, were set to the number of features / 3 and 500 respectively according to the recommended values.

## Supplementary information

**Additional file 1.** **Table S1.** The variable importance scores of all 1952 features.

**Additional file 2.** **Table S2.** The prediction accuracy of all random forest models on different training samples.

**Additional file 3.** **Table S3.** AUCs and AUPRs of the RFLDA in each cross-validation.

**Additional file 4.** **Table S4.** The predicted top 50 lncRNAs associated with all 412 diseases by the RFLDA.

## Data Availability

The following are available online: Table S1: The variable importance scores of all 1952 features. Table S2: The prediction accuracy of all random forest models on different training samples. Table S3: AUCs and AUPRs of the RFLDA in each cross-validation. Table S4: The predicted top 50 lncRNAs associated with all 412 diseases by the RFLDA. The original data and code of RFLDA is available at: https://github.com/ydkvictory/RFLDA

## Abbreviations

lncRNA: Long non-coding RNA
LDA: lncRNA-disease association
RF: Random forest
MDA: miRNA-disease association
DSS: Disease semantic similarity
LFS: lncRNA functional similarity
LMI: lncRNA-miRNA interactions
AUC: The area under the receiver operating characteristic curve
AUPR: The area under the precision-recall curve
RWR: Random walk with restart
DAG: Directed acyclic graph
GIPKS: Gaussian interaction profile kernel similarity
FPR: False positive rate
TPR: True positive rate
TP: True positive
FN: False negative
FP: False positive
TN: True negative
ROC: Receiver operating characteristic
DO: Disease ontology
OOB: Outside of bag

## References

[1] Ponting CP, Oliver PL, Reik W (2009). Evolution and functions of long noncoding RNAs. Cell.

[2] Lu Q, Ren S, Lu M, Zhang Y, Zhu D, Zhang X, Li T (2013). Computational prediction of associations between long non-coding RNAs and proteins. BMC Genomics.

[3] Li J, Xuan Z, Liu C (2013). Long non-coding RNAs and complex human diseases. Int J Mol Sci.

[4] Chen X, Sun YZ, Guan NN, Qu J, Huang ZA, Zhu ZX, Li JQ (2019). Computational models for lncRNA function prediction and functional similarity calculation. Brief Funct Genomics.

[5] Chen X, Yan CC, Zhang X, You ZH (2017). Long non-coding RNAs and complex diseases: from experimental results to computational models. Brief Bioinform.

[6] Zhang X, Zhou Y, Mehta KR, Danila DC, Scolavino S, Johnson SR, Klibanski A (2003). A pituitary-derived MEG3 isoform functions as a growth suppressor in tumor cells. J Clin Endocrinol Metab.

[7] Faghihi MA, Modarresi F, Khalil AM, Wood DE, Sahagan BG, Morgan TE, Finch CE, Laurent GS, Kenny PJ, Wahlestedt C (2008). Expression of a noncoding RNA is elevated in Alzheimer's disease and drives rapid feed-forward regulation of β-secretase. Nat Med.

[8] Congrains A, Kamide K, Oguro R, Yasuda O, Miyata K, Yamamoto E, Kawai T, Kusunokif H, Yamamoto H, Takeya Y, Yamamoto K, Onishia M, Sugimoto K, Katsuya T, Awata N, Ikebe K, Gondo Y, Oike Y, Ohishi M, Rakugi H (2012). Genetic variants at the 9p21 locus contribute to atherosclerosis through modulation of ANRIL and CDKN2A/B. Atherosclerosis.

[9] Johnson R (2012). Long non-coding RNAs in Huntington's disease neurodegeneration. Neurobiol Dis.

[10] Sun J, Shi HB, Wang ZZ, Zhang CJ, Liu L, Wang LT, He WW, Hao DP, Liu SL, Zhou M (2014). Inferring novel lncRNA–disease associations based on a random walk model of a lncRNA functional similarity network. Mol BioSyst.

[11] Zhou M, Wang XJ, Li JW, Hao DP, Wang ZZ, Shi HB, Han L, Zhou H, Sun J (2015). Prioritizing candidate disease-related long non-coding RNAs by walking on the heterogeneous lncRNA and disease network. Mol BioSyst.

[12] Chen X (2015). KATZLDA: KATZ measure for the lncRNA-disease association prediction. Sci Rep.

[13] Chen X, You ZH, Yan GY, Gong DW (2016). IRWRLDA: improved random walk with restart for lncRNA-disease association prediction. Oncotarget.

[14] Yu GX, Fu GY, Lu C, Ren Y, Wang J (2017). BRWLDA: bi-random walks for predicting lncRNA-disease associations. Oncotarget.

[15] Gu CL, Liao B, Li XY, Cai LJ, Li ZJ, Li KQ, Yang JL (2017). Global network random walk for predicting potential human lncRNA-disease associations. Sci Rep.

[16] Zhang J, Zhang Z, Chen Z, Deng L (2017). Integrating multiple heterogeneous networks for novel lncRNA-disease association inference. IEEE/ACM Trans Comput Biol Bioinform.

[17] Xiao XF, Zhu W, Liao B, Xu JL, Gu CL, Ji BB, Yao YH, Peng LH, Yang JL (2018). BPLLDA: predicting lncRNA-disease associations based on simple paths with limited lengths on a heterogeneous network. Front Genet.

[18] Ping PY, Wang L, Kuang LN, Ye ST, Iqbal MFB, Pei TR (2019). A novel method for lncRNA-disease association prediction based on an lncRNA-disease association network. IEEE/ACM Trans Comput Biol Bioinform.

[19] Fan XN, Zhang SW, Zhang SY, Zhu K, Lu S (2019). Prediction of lncRNA-disease associations by integrating diverse heterogeneous information sources with RWR algorithm and positive pointwise mutual information. BMC Bioinformatics.

[20] Liu Y, Feng X, Zhao HC, Xuan ZW, Wang L (2019). A novel network-based computational model for prediction of potential LncRNA-disease association. Int J Mol Sci.

[21] Li JC, Zhao HC, Xuan ZW, Yu JW, Feng X, Liao B, Wang L (2019). A novel approach for potential human LncRNA-disease association prediction based on local random walk. IEEE/ACM Trans. Comput. Biol. Bioinform.

[22] Sumathipala M, Maiorino E, Weiss ST, Sharma A (2019). Network diffusion approach to predict lncRNA disease associations using multi-type biological networks: LION. Front Physiol.

[23] Zhang H, Liang YC, Peng C, Han SY, Du W, Li Y (2019). Predicting lncRNA-disease associations using network topological similarity based on deep mining heterogeneous networks. Math Biosci.

[24] Xie GB, Meng TF, Luo Y, Liu ZG (2019). SKF-LDA: similarity kernel fusion for predicting lncRNA-disease association. Ther-Nucl Acids.

[25] Liu MX, Chen X, Chen G, Cui QH, Yan GY (2014). A computational framework to infer human disease-associated long noncoding RNAs. PLoS One.

[26] Li JW, Gao C, Wang YC, Ma W, Tu J, Wang JP, Chen ZZ, Kong W, Cui QH (2014). A bioinformatics method for predicting long noncoding RNAs associated with vascular disease. Sci China Life Sci.

[27] Chen X (2015). Predicting lncRNA-disease associations and constructing lncRNA functional similarity network based on the information of miRNA. Sci Rep.

[28] Wang JY, Ma RX, Ma W, Chen J, Yang JC, Xi YG, Cui QH (2016). LncDisease: a sequence based bioinformatics tool for predicting lncRNA-disease associations. Nucleic Acids Res.

[29] Cheng L, Shi HB, Wang ZZ, Hu Y, Yang HX, Zhou C, Sun J, Zhou M (2016). IntNetLncSim: an integrative network analysis method to infer human lncRNA functional similarity. Oncotarget.

[30] Wang P, Guo QY, Gao Y, Zhi H, Zhang Y, Liu Y, Zhang JZ, Yue M, Guo MN, Ning SW, Zhang GM, Li X (2017). Improved method for prioritization of disease associated lncRNAs based on ceRNA theory and functional genomics data. Oncotarget.

[31] Fu GY, Wang J, Domeniconi C, Yu GX (2018). Matrix factorization-based data fusion for the prediction of lncRNA–disease associations. Bioinformatics.

[32] Ding L, Wang MH, Sun DD, Li A (2018). TPGLDA: novel prediction of associations between lncRNAs and diseases via lncRNA-disease-gene tripartite graph. Sci Rep.

[33] Lu CQ, Yang MY, Luo F, Wu FX, Li M, Pan Y, Li YH, Wang JX (2018). Prediction of lncRNA–disease associations based on inductive matrix completion. Bioinformatics.

[34] Wang YH, Yu GX, Wang J, Fu GY, Guo MZ, Domeniconi C (2020). Weighted matrix factorization on multi-relational data for LncRNA-disease association prediction. Methods.

[35] Chen X, Yan GY (2013). Novel human lncRNA-disease association inference based on lncRNA expression profiles. Bioinformatics.

[36] Chen X, Yan CC, Luo C, Ji W, Zhang Y, Dai Q (2015). Constructing lncRNA functional similarity network based on lncRNA-disease associations and disease semantic similarity. Sci Rep.

[37] Huang YA, Chen X, You ZH, Huang DS, Chan KC (2016). ILNCSIM: improved lncRNA functional similarity calculation model. Oncotarget.

[38] Zhao TT, Xu JY, Liu L, Bai J, Xu CH, Xiao Y, Li X, Zhang LM (2015). Identification of cancer-related lncRNAs through integrating genome, regulome and transcriptome features. Mol BioSyst.

[39] Lan W, Li M, Zhao KJ, Liu J, Wu FX, Pan Y, Wang JX (2017). LDAP: a web server for lncRNA-disease association prediction. Bioinformatics.

[40] Yu JW, Xuan ZW, Feng X, Zou Q, Wang L (2019). A novel collaborative filtering model for LncRNA-disease association prediction based on the Naïve Bayesian classifier. BMC Bioinformatics.

[41] Guo ZH, You ZH, Wang YB, Yi HC, Chen ZH (2019). A Learning-Based Method for LncRNA-Disease Association Identification Combing Similarity Information and Rotation Forest. iScience.

[42] Chen QF, Lai DH, Lan W, Wu XM, Chen BS, Chen YPP, Wang JX (2019). ILDMSF: Inferring Associations between Long non-coding RNA and Disease Based on Multi-similarity Fusion. IEEE/ACM Trans. Comput. Biol. Bioinform.

[43] Guo ZH, Yi HC, You ZH (2019). Construction and comprehensive analysis of a molecular association network via lncRNA-miRNA-disease-drug-protein graph. Cells.

[44] Xuan P, Cao YK, Zhang TG, Kong R, Zhang ZG (2019). Dual convolutional neural networks with attention mechanisms based method for predicting disease-related lncRNA genes. Front Genet.

[45] Xuan P, Pan SX, Zhang TG, Liu Y, Sun H (2019). Graph convolutional network and convolutional neural network based method for predicting lncRNA-disease associations. Cells.

[46] Xuan P, Sheng N, Zhang TG, Liu Y, Guo YH (2019). CNNDLP: a method based on convolutional autoencoder and convolutional neural network with adjacent edge attention for predicting lncRNA–disease associations. Int J Mol Sci.

[47] Xuan P, Jia L, Zhang TG, Sheng N, Li XK, Li JB (2019). LDAPred: a method based on information flow propagation and a convolutional neural network for the prediction of disease-associated lncRNAs. Int J Mol Sci.

[48] Chen X, Wang CC, Yin J, You ZH (2018). Novel human miRNA-disease association inference based on random forest. Mol Ther-Nucl Acids.

[49] Hajian-Tilaki K (2013). Receiver operating characteristic (ROC) curve analysis for medical diagnostic test evaluation. Caspian J Intern Med.

[50] Saito T, Rehmsmeier M (2015). The precision-recall plot is more informative than the ROC plot when evaluating binary classifiers on imbalanced datasets. PLoS One.

[51] Ning SW, Zhang JZ, Wang P, Zhi H, Wang JJ, Liu Y, Gao Y, Guo MN, Yue M, Wang LH, Li X (2016). Lnc2Cancer: a manually curated database of experimentally supported lncRNAs associated with various human cancers. Nucleic Acids Res.

[52] Chen G, Wang ZY, Wang DQ, Qiu CX, Liu MX, Chen X, Zhang QP, Yan GY, Cui QH (2013). LncRNADisease: a database for long-non-coding RNA-associated diseases. Nucleic Acids Res.

[53] Bahari F, Emadi-Baygi M, Nikpour P (2015). miR-17-92 host gene, uderexpressed in gastric cancer and its expression was negatively correlated with the metastasis. Ind J Cancer.

[54] Zhang J, Lu S, Zhu JF, Yang KP (2016). Up-regulation of lncRNA HULC predicts a poor prognosis and promotes growth and metastasis in non-small cell lung cancer. Int J Clin Exp Pathol.

[55] Cheng DZ, Bao CC, Zhang XX, Lin XS, Huang HO, Zhao L (2018). LncRNA PRNCR1 interacts with HEY2 to abolish miR-448-mediated growth inhibition in non-small cell lung cancer. Biomed Pharmacother.

[56] Xu J, Meng QT, Li XB, Yang HB, Xu J, Gao N, Sun H, Wu SS, Familiari G, Relucenti M, Zhu HT, Wu J, Chen R (2019). Long noncoding RNA MIR17HG promotes colorectal Cancer progression via miR-17-5p. Cancer Res.

[57] Chen X, Wang L, Qu J, Guan NN, Li JQ (2018). Predicting miRNA–disease association based on inductive matrix completion. Bioinformatics.

[58] Chen X, Xie D, Zhao Q, You ZH (2019). MicroRNAs and complex diseases: from experimental results to computational models. Brief Bioinform.

[59] Gao YL, Cui Z, Liu JX, Wang J, Zheng CH (2019). NPCMF: nearest profile-based collaborative matrix factorization method for predicting miRNA-disease associations. BMC Bioinformatics.

[60] Chen X, Yin J, Qu J, Huang L (2018). MDHGI: matrix decomposition and heterogeneous graph inference for miRNA-disease association prediction. PLoS Comput Biol.

[61] Yin MM, Cui Z, Gao MM, Liu JX, Gao YL (2019). LWPCMF: logistic weighted profile-based collaborative matrix factorization for predicting MiRNA-disease associations. IEEE/ACM Trans. Comput. Biol. Bioinform.

[62] Cui Z, Liu JX, Gao YL, Zhu R, Yuan SS (2019). LncRNA-disease associations prediction using bipartite local model with nearest profile-based association inferring. IEEE J Biomed Health Inform.

[63] Chen X, Yan CC, Zhang X, Zhang X, Dai F, Yin J, Zhang Y (2016). Drug–target interaction prediction: databases, web servers and computational models. Brief Bioinform.

[64] Chen X, Ren B, Chen M, Wang Q, Zhang L, Yan G (2016). NLLSS: predicting synergistic drug combinations based on semi-supervised learning. PLoS Comput Biol.

[65] Lu ZY, Coben KB, Hunter L (2007). GeneRIF quality assurance as summary revision. Biocomputing.

[66] Li Y, Qiu CX, Tu J, Geng B, Yang JC, Jiang TZ, Cui QH (2014). HMDD v2. 0: a database for experimentally supported human microRNA and disease associations. Nucleic acids Res.

[67] Li JH, Liu S, Zhou H, Qu LH, Yang JH (2014). starBase v2. 0: decoding miRNA-ceRNA, miRNA-ncRNA and protein-RNA interaction networks from large-scale CLIP-Seq data. Nucleic Acids Res.

[68] Kibbe WA, Arze C, Felix V, Mitraka E, Bolton E, Fu G, Mungall CJ, Binder JX, Malone J, Vasant D, Parkinson H, Schriml LM (2015). Disease Ontology 2015 update: an expanded and updated database of human diseases for linking biomedical knowledge through disease data. Nucleic Acids Res.

[69] Wang JZ, Du Z, Payattakool R, Yu PS, Chen CF (2007). A new method to measure the semantic similarity of GO terms. Bioinformatics.

[70] Cheng L, Hu Y, Sun J, Zhou M, Jiang QH (1953). DincRNA: a comprehensive web-based bioinformatics toolkit for exploring disease associations and ncRNA function. Bioinformatics.

[71] Yang GD, Lu XZ, Yuan LJ (2014). LncRNA: a link between RNA and cancer. Biochim et Biophys Acta.

[72] Breiman L (2001). Random forests. Mach Learn.

[73] Verikas A, Gelzinis A, Bacauskiene M (2011). Mining data with random forests: a survey and results of new tests. Pattern Recogn.

[74] Nimrod G, Szilágyi A, Leslie C, Ben-Tal N (2009). Identification of DNA-binding proteins using structural, electrostatic and evolutionary features. J Mol Biol.

[75] Heidema AG, Boer JM, Nagelkerke N, Mariman EC, Feskens EJ (2006). The challenge for genetic epidemiologists: how to analyze large numbers of SNPs in relation to complex diseases. BMC Genet.

[76] Han P, Zhang X, Norton RS, Feng ZP (2009). Large-scale prediction of long disordered regions in proteins using random forests. BMC Bioinformatics.

[77] Yao DJ, Yang J, Zhan XJ, Zhan XR, Xie ZQ (2015). A novel random forests-based feature selection method for microarray expression data analysis. Int J Data Min Bioin.

[78] Yao DJ, Zhan XJ, Kwoh CK (2019). An improved random forest-based computational model for predicting novel miRNA-disease associations. BMC Bioinformatics.

